# Digital markers of sleep architecture to characterize the impact of different lockdown regimens on sleep health during the COVID-19 pandemic

**DOI:** 10.1093/sleep/zsac074

**Published:** 2022-04-16

**Authors:** Jean-Louis Pépin, Sébastien Bailly, Emmanuel Mignot, Jonathan Gaucher, Alexandre Chouraki, Mallory Cals-Maurette, Raoua Ben Messaoud, Renaud Tamisier, Pierrick J Arnal

**Affiliations:** Laboratoire Hypoxie et Physiopathologies cardiovasculaires et respiratoires, HP2 Laboratory, INSERM U1300, University Grenoble Alpes, Grenoble, France; EFCR Laboratory, Grenoble Alpes University Hospital, Grenoble, France; Laboratoire Hypoxie et Physiopathologies cardiovasculaires et respiratoires, HP2 Laboratory, INSERM U1300, University Grenoble Alpes, Grenoble, France; EFCR Laboratory, Grenoble Alpes University Hospital, Grenoble, France; Center for Sleep Sciences and Medicine, Stanford University, Palo Alto, CA, USA; Laboratoire Hypoxie et Physiopathologies cardiovasculaires et respiratoires, HP2 Laboratory, INSERM U1300, University Grenoble Alpes, Grenoble, France; EFCR Laboratory, Grenoble Alpes University Hospital, Grenoble, France; Science Team, Dreem SAS, Paris, France; Laboratoire Hypoxie et Physiopathologies cardiovasculaires et respiratoires, HP2 Laboratory, INSERM U1300, University Grenoble Alpes, Grenoble, France; EFCR Laboratory, Grenoble Alpes University Hospital, Grenoble, France; Laboratoire Hypoxie et Physiopathologies cardiovasculaires et respiratoires, HP2 Laboratory, INSERM U1300, University Grenoble Alpes, Grenoble, France; EFCR Laboratory, Grenoble Alpes University Hospital, Grenoble, France; Laboratoire Hypoxie et Physiopathologies cardiovasculaires et respiratoires, HP2 Laboratory, INSERM U1300, University Grenoble Alpes, Grenoble, France; EFCR Laboratory, Grenoble Alpes University Hospital, Grenoble, France; Science Team, Dreem SAS, Paris, France

Dear Editor,

Changes in behaviors during lockdowns implemented to prevent the spread of COVID-19 impacted sleep schedules and sleep health. Home confinement was associated with escalations in stress and anxiety, and the restriction of social activities all contributed to the exacerbation of mental disorders, intrafamilial conflicts, and addictions [1]. During lockdowns, there was a loss of external synchronizers such as regular mealtimes; and physical activity was reduced [2]. The loss in regularity in these crucial circadian timekeepers impacted sleep schedules, duration, and quality, and contributed to other confinement-related effects on sleep. The deterioration of sleep health during lockdowns has mainly been studied using questionnaires or online surveys, with the acknowledged methodological concerns and subjectivity [3–5]. Few studies have objectively characterized changes in sleep macro- and microarchitecture during lockdowns [6, 7]. Our team previously collected objective sleep data, via a sleep-monitoring headband, over several weeks before and after the COVID-19 total lockdown in spring 2020 in France [6]. Briefly, we demonstrated that during total lockdown, individuals slept more, had less deep sleep, more light sleep, and longer rapid eye movement (REM) sleep [6]. Most studies were conducted during the first wave of COVID-19 in early 2020 [8]. However, the persistence of abnormal sleep patterns after reopening and specific sleep modifications related to less strict confinement regimens remain poorly elucidated. Quickly after the lifting of restrictions increased physical activity was observed [2], alongside in-person return to work and schools reopening. This realignment of the majority of social synchronizers might potentially trigger a return to usual bedtimes and wake-up times, and consolidate sleep cycles, thus improving sleep quality [7]. It is of major importance to better understand the kinetics of changes in sleep patterns across lockdowns so as to better guide health policies and rapidly implement tailored interventions when sleep perturbations persist in the mid to long term. To address these issues, we analyzed objective data on sleep macro- and microarchitecture repeatedly collected over multiple nights before, during, and after three consecutive COVID-19 French government-imposed lockdowns.

During the first 8-week total lockdown (March 17–May 10, 2020) strict measures were deployed to abate the spread of COVID-19. From mid-May restrictions were gradually relaxed. The second-wave curfew (October 23–October 29, 2020) was followed by a partial lockdown (October 30–December 15, 2020) that was less strict. Freedom of movement within 10 km facilitated social contacts and out-of-door physical activity, and parks and gardens stayed open. More businesses remained open with more sectors authorized to continue working in-person, including factories. School closures were less frequent with less workers staying at home involuntarily. Working from home was recommended but at least 1 day per week attendance at the workplace was mandatory.

The sleep-monitoring device (Dreem SAS, Paris) is a wireless headband with five dry EEG electrodes yielding seven EEG derivation. It records, stores, and automatically analyzes objective sleep data in real time [6, 9]. A robust validation of automatic sleep staging using the device has been made with performances similar to the average of five sleep scoring experts [10]. The sleep-monitoring headband allows repeated measurement of sleep architecture, i.e. sleep latency (sleep onset duration [SOL]), total sleep time (TST), and the duration of sleep stages (N2, N3, and REM).

The sleep-monitoring wellness device is sold directly to the consumer. Thus, Institutional Review Board ethical approval was not sought for our secondary analyses of previously collected data of Dreem headband customers. All included individuals had given their informed consent for the use of their pseudonymized data for research purposes. Sleep data recorded during 2019 in the same individuals were used as control.

Data are reported as median and interquartile range [Q1; Q3] for quantitative values and as numbers and percentage for qualitative values. Generalized linear mixed models, with a participant random effect and including interaction terms such as year and period were used to assess the independent effects of lockdown periods on sleep parameters. Statistical analyses were performed by using SAS v9.4 (SAS Institute Inc., Cary, NC) and R v4.1.1 software (R Foundation for Statistical Computing, Vienna, Austria). A *p* value of less than 0.05 was considered as statistically significant.

The 599 study participants were regular French users of the Dreem headband throughout 2019 and 2020. They were mainly men (71%) with a median age of 47 years (36–59). Some exhibited a primary morningness (23%) or eveningness (18.4%) chronotype. Moderate-to-severe symptoms of anxiety (HADS-A >10/21) were self-reported by 18.7% of participants, and moderate-to-severe symptoms of insomnia (ISI ≥10/28) were reported by 61%.

Figure 1 shows changes in sleep schedules during the different lockdown regimens. For the overall study cohort, significant and progressive shifts in bedtime and wake-up time were observed only during total lockdown. No significant change was found during partial lockdown and curfew periods. After resumption of social and economic activities, return to similar sleep schedules to those during the corresponding period in 2019 was achieved within 2 weeks. For individuals with an eveningness chronotype there was a trend in the same direction as changes during total lockdown, but significance was not achieved.

**Figure 1.**
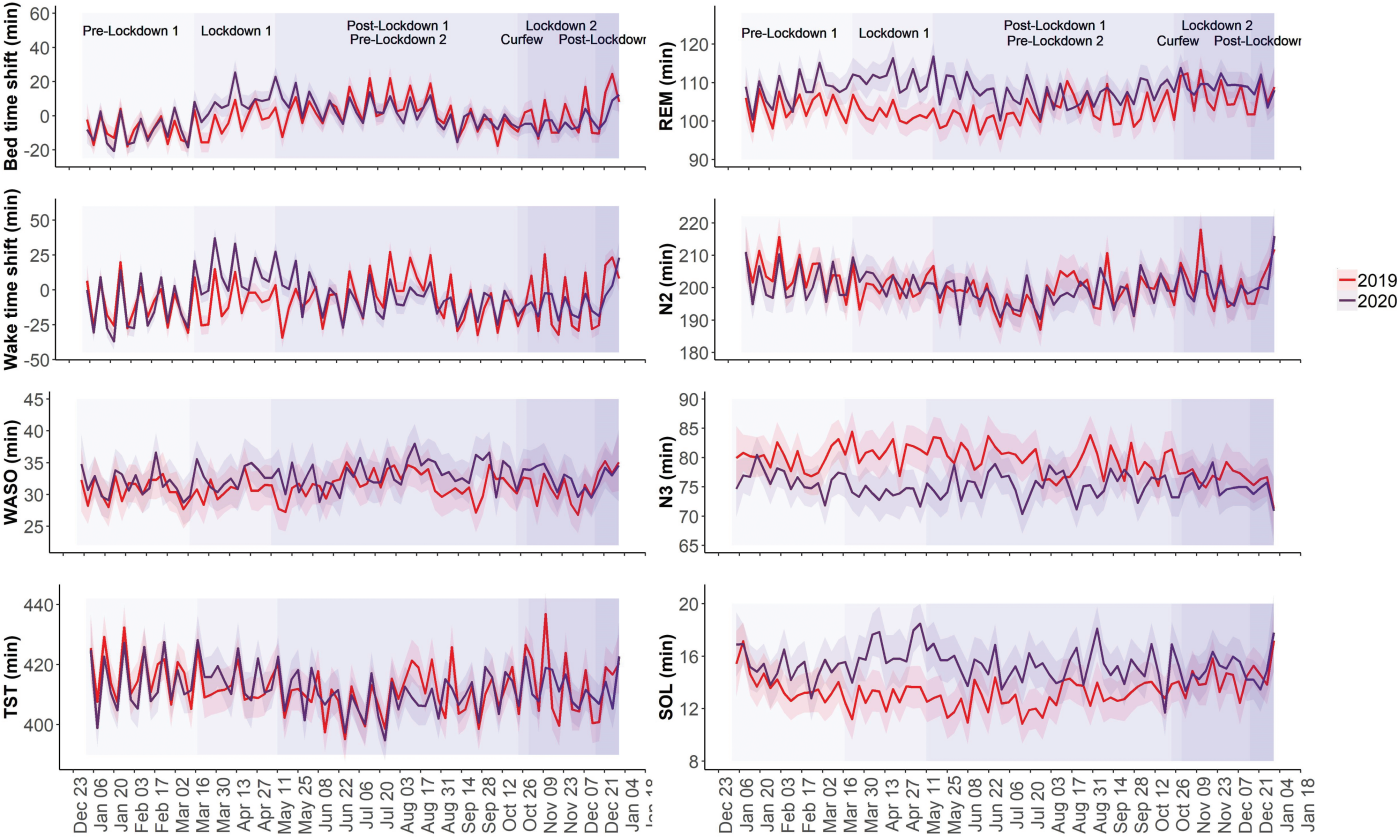
Sleep schedules and sleep architecture before and during the different lockdown regimens. The shading above and below the lines represent the 95% confidence intervals. N2, light sleep; N3, deep sleep; WASO, wake-time-after-sleep-onset.

During the first total lockdown there was a significant change in sleep architecture with an increase in TST, getting to sleep (SOL), and light sleep time (N2) [6]. We found no significant change in wake-time-after-sleep-onset and sleep efficiency. However, during the second partial lockdown and curfew, using year 2019 as control, no significant change in sleep architecture was found (Table 1). Again, return to similar characteristics as in 2019 was achieved in 2–3 weeks.

**Table 1.** Objective sleep parameters

	Pre Lockdown 1 (reference)	Lockdown 1	Post Lockdown 1	Curfew	Lockdown 2	Post Lockdown 2
Sleep schedule						
TST (min)	415 [371; 457]	420 [371; 457]	411 [367; 453]	417 [375; 464]	414 [369; 455]	411 [366; 154]
		7.38 [2.8; 11.9]	2.81 [−3.1; 8.7]	3.05 [−6.5; 12.6]	2.83 [−5.1; 10.8]	0.34 [−8.7; 9.4]
		**0.0015**	0.3476	0.5288	0.4847	0.9398
WASO (min)	20 [12; 37]	21 [12; 38]	22 [13; 40]	21 [13; 40]	21 [12; 39]	21 [12; 40]
		0.80 [−1.2; 2.9]	0.63 [−2; 3.3]	1.52 [−3.1; 6.2]	1.04 [−2.5; 4.6]	−2.85 [−7.5; 1.8]
		0.4407	0.6353	0.5200	0.5676	0.2260
Sleep architecture						
N2 (min)	199 [165; 235]	202 [168; 238]	197 [163; 231]	200 [169; 236]	198 [162; 232]	199 [165; 235]
		6.31 [2.5; 10.1]	2.75 [−2.3; 7.8]	4.93 [−2.7; 12.6]	2.05 [−4.6; 8.7]	2.67 [−5; 10.3]
		**0.0012**	0.2853	0.2040	0.5442	0.4926
N3 (min)	75 [53; 97]	72 [51; 95]	75 [52; 96]	71 [51; 93]	74 [54; 96]	74 [53; 94]
		−3.63 [−6.3; −1]	−0.68 [−4.4; 3]	−2.51 [−8; 3]	2.40 [−2.5; 7.3]	2 [−3.2; 7.2]
		**0.0070**	0.7152	0.3704	0.3319	0.4491
REM (min)	106 [83; 129]	108 [86; 132]	105 [83; 129]	107 [87; 133]	107 [84; 129]	104 [83; 129]
		4.34 [1.7; 7]	0.52 [−2.6; 3.7]	0.24 [−5; 5.5]	−1.72 [−6.4; 2.9]	−3.14 [−8.3; 2]
		**0.0012**	0.7413	0.9271	0.4668	0.2328
SOL (min)	10 [6; 18]	11 [6; 19]	9 [5; 17]	10 [6; 19]	10 [6; 17]	10 [6; 17]
		2.08 [1.1; 3.1]	0.84 [−0.7; 2.4]	0.94 [−1.5; 3.4]	−0.91 [−3.2; 1.3]	−1.79 [−4; 0.4]
		**<0.0001**	0.2827	0.4433	0.4278	0.1119

All values are given as median [*Q*1; *Q*3], and estimate [95% CI] with *p* value (in bold if statistically significant). N2, light sleep; N3, deep sleep; WASO, wake-time-after-sleep-onset.

Digital markers of sleep schedules and sleep architecture can be used to characterize the impact of different regimens of social restrictions and the time course of recovery during the COVID-19 pandemic. A major strength of our data is the use of the previous year, 2019, as a control period allowing us to assume that the major confounders of variations in sleep patterns were accounted for. Briefly, we demonstrated that the total strict lockdown affected sleep schedules and sleep quality, but these alterations did not occur when people had the possibility of person-to-person social activities, albeit restricted, and were able to do some physical exercise. The digital technology used in this study offers several advantages over conventional subjective assessments. First, it provides objective and robust recording of sleep patterns in ecological conditions since the headband is worn in the subject’s home environment. Second, repeated, whole night assessments can be implemented over periods of several months or years with little cost. Third, scale-up is straightforward and would permit the assessment of hundreds even thousands of people simultaneously, providing population representative whole population data.

Our observations indicate that relaxing lockdown mitigates the risk of deterioration in sleep health. This provides the scientific community and policymakers with data to gauge the risk/benefit ratio of the imposition of a legitimate reduction in social contacts to minimize virus propagation.

## Data Availability

The data that support the findings of this study are available on reasonable request to the corresponding author

## Code Availability

The code that supports the findings of this study are available on reasonable request to the corresponding author.

## References

[1] Spinelli A, et al COVID-19 lockdowns and incidence of psychoactive substance exposure according to age and sex. Clin Toxicol (Phila). 2022;60:596–601. 34904494 10.1080/15563650.2021.2013494

[2] Pépin JL, et al Wearable activity trackers for monitoring adherence to home confinement during the COVID-19 pandemic worldwide: data aggregation and analysis. J Med Internet Res. 2020;22(6):e19787. 32501803 10.2196/19787 PMC7307323

[3] Bottary R, et al Age and chronotype influenced sleep timing changes during the first wave of the COVID-19 pandemic. J Sleep Res. 2021;2:e13495. 10.1111/jsr.13495 PMC8646670 34608693

[4] O’Regan D, et al Understanding the impact of the COVID-19 pandemic, lockdowns and social isolation on sleep quality. Nat Sci Sleep. 2021;13:2053–2064. 34795545 10.2147/NSS.S266240 PMC8593898

[5] Pérez-Carbonell L, et al Impact of the novel coronavirus (COVID-19) pandemic on sleep. J Thorac Dis. 2020;12(Suppl 2):S163–S175. 33214921 10.21037/jtd-cus-2020-015 PMC7642637

[6] Pépin JL, et al Greatest changes in objective sleep architecture during COVID-19 lockdown in night owls with increased REM sleep. Sleep. 2021;44(9). doi:10.1093/sleep/zsab075 PMC8083638 33769511

[7] Massar SAA, et al Reopening after lockdown: the influence of working-from-home and digital device use on sleep, physical activity, and wellbeing following COVID-19 lockdown and reopening. Sleep. 2022;45(1). doi:10.1093/sleep/zsab250 PMC8549292 34636396

[8] Conte F, et al High sleep fragmentation parallels poor subjective sleep quality during the third wave of the Covid-19 pandemic: an actigraphic study. J Sleep Res. 2021:e13519. 34797004 10.1111/jsr.13519 PMC8646572

[9] Debellemaniere E, et al Performance of an ambulatory dry-EEG device for auditory closed-loop stimulation of sleep slow oscillations in the home environment. Front Hum Neurosci. 2018;12:88. 29568267 10.3389/fnhum.2018.00088 PMC5853451

[10] Arnal PJ, et al The Dreem Headband compared to polysomnography for electroencephalographic signal acquisition and sleep staging. Sleep. 2020;43(11). doi:10.1093/sleep/zsaa097 PMC7751170 32433768

